# Congenital single kidney in tuberous sclerosis complex (Bourneville disease)

**DOI:** 10.1097/MS9.0000000000001834

**Published:** 2024-02-20

**Authors:** Wafaa Bzeih, Mohammad Kbar, Oussama Rihan

**Affiliations:** a Faculty of Medical Sciences, Lebanese University, Hadath; b Bahman Hospital, Beirut, Lebanon

**Keywords:** Case report, tuberous sclerosis, bourneville disease, renal angiolipomatosis, single kidney

## Abstract

**Introduction:**

Bournevile disease is a rare global condition that presents a diagnostic challenge due to its diverse multisystemic involvement.

**Case presentation:**

This report presents the case of a 14-year-old male with a medical history of congenital single kidney, heart rate abnormalities, Bournevile disease with cognitive deficits, brain lesions, and dermatological features. The patient presented with sudden onset renal angiolipomatosis, and the diagnosis was based on specific computed tomography (CT) findings. Despite having these complex medical conditions, the patient had never been admitted to the hospital since infancy, and treatment was limited to surveillance only.

**Clinical discussion:**

Bourneville disease is a multisystemic disease that affects several organ systems within the human body and thus demands multidisciplinary approach in the treatment and follow-up options.

**Conclusion:**

This case report highlights Bournevile disease in a patient with a congenital single kidney, a rare finding that further complicates the disease. It emphasises the importance of recognising and managing this condition to ensure appropriate care for affected individuals.

## Background

Highlights

- Tuberous sclerosis complex (TSC) is a rare neurocutaneous syndrome that affects multiple organs, including the skin, brain, kidneys, heart, and lungs. The case emphasises the systemic involvement of various organs in TSC.
- The patient mentioned in the case is a 14-year-old boy with TSC, congenital single kidney, cognitive deficits, brain lesions, and dermatological features. The diagnosis of TSC was made at age two due to seizures, with EEG and MRI confirming TSC.
- Kidney assessment in 2010 revealed a normal left kidney, but recent imaging showed nodular lesions with fat density typical of angiolipomatosis. The patient presented with flank pain, and imaging showed the enlarged left kidney with angiolipomatosis.
- This case is notable for the absence of hospitalisation despite acute renal findings in a patient with a congenital single kidney, underscoring the importance of surveillance and adaptive kidney function.
- Early diagnosis, coordinated multidisciplinary care, and regular surveillance are crucial for managing TSC effectively.

The neurocutaneous syndrome known as tuberous sclerosis or Bourneville disease affects the skin and other internal organs including the kidneys, eyes, heart, brain, and lungs. Thus, as an emphasis on the multiorgan systemic involvement, the enlarged phenotype of tuberous sclerosis is now referred to as the “tuberous sclerosis complex” which has a frequency of 1 in 5000–10 000 live births^1^.

The most frequent findings in TSC are cutaneous, which are considered initial indicators for the diagnosis in most of the cases^1^. In addition, 85% of tuberous sclerosis complex (TSC) patients suffer from epilepsy, which manifests in the first year of life^2^. Children presenting with epilepsy often possess cortical tubers which are referred to as glioneuronal hamartomas, subependymal nodules, and subependymal giant cell astrocytomas^2^. However, hamartomas are not limited to the brain as they can also affect the heart and the kidneys too^2,3^. Furthermore, at least 50% of patients experience cognitive impairments and learning difficulties^4^.

The diagnosis of TSC is primarily clinical, based on the diagnostic criteria of major and/or minor characteristics in addition to neuroradiological imaging modalities^5^. Epilepsy, intellectual disability, and the presence of adenoma sebaceum make up the typical clinical diagnostic triad that results from the association of the cerebral, renal, cardiac, and cutaneous manifestations^6^. Once the diagnosis is confirmed, multiple specialties should collaborate to manage the condition, including regular imaging follow-ups and symptomatic care^7^. Brain tests, including electroencephalograms, retinal and skin examinations, evaluate brain function; treatments like excision, dermabrasion, and laser control symptoms^7^.

TSC is a globally rare disease which hasn’t been reported in the literature in the Lebanese population especially that our patient presents with a congenital single kidney.

## Case presentation

This is a case of a 14-year-old Arab boy, non-smoker, with no known food or drug allergies, known to have congenital single kidney and tuberous sclerosis with cognitive deficits, brain lesions, and dermatological features. Initially, the boy was diagnosed prenatally with heart rate abnormalities (bradycardia) and a single kidney. At the age of two years, the boy started having multiple myoclonic seizures treated with levetiracetam in which EEG was performed by a neurologist along with an MRI study which confirmed the presence of cortical and subcortical hamartomas in the brain and led to the diagnosis of tuberous sclerosis.

During the course of the 14 years, the boy was never admitted to hospital for any serious complication but developed mental retardation and typical skin manifestations which include facial angiofibromas and hypomelanotic macules. Kidney morphological features were assessed in 2010 by Kidney US and confirmed normal left kidney with normal cortical thickness and good corticomedullary differentiation. The seizures were controlled using Sodium Valproate and Levetiracetam, with regular creatinine checkups that never showed a value of creatinine clearance more than one, and annual follow-up brain MRIs were done which confirmed typical findings of subtentorial subcortical and cortical hamartomas in addition to subependymal lesions suggestive of benign giant cell tumour.

**Figure 1:**
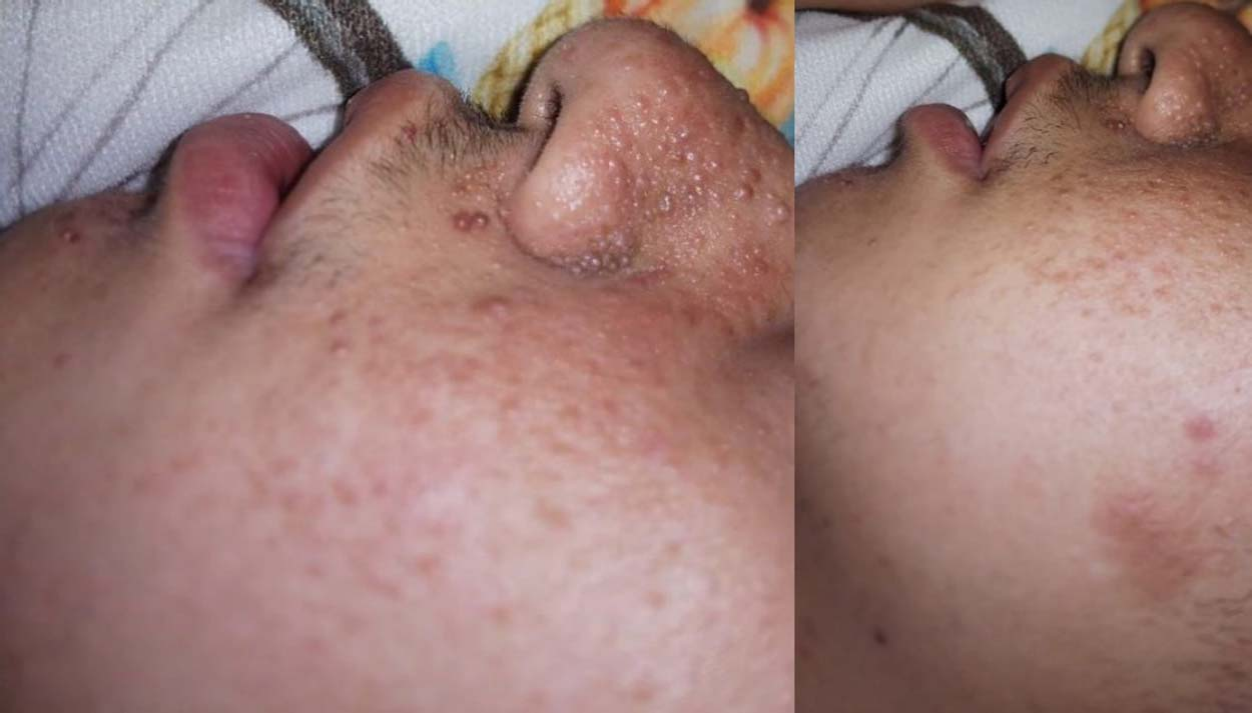
Picture showing small molluscums and pigmented plaques on the face of the child especially region around the nose and cheek.

**Figure 2:**
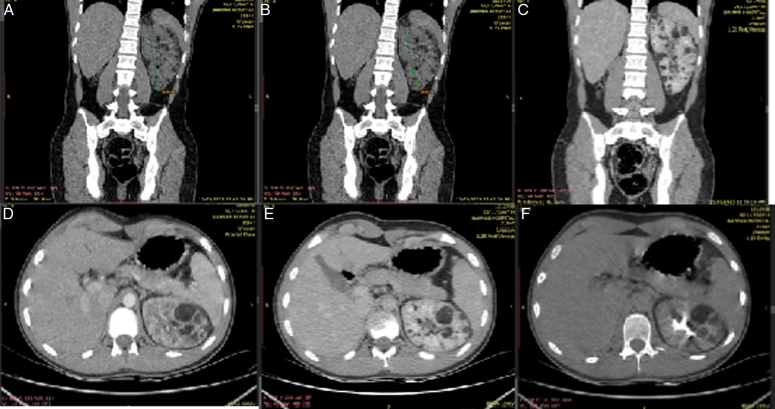
Several views of the abdomino-pelvic computed tomography scan showing a single left kidney presenting with angiolipomatosis [(A, B) being plain coronal without contrast showing enlarged kidney dimensions with a longitudinal diameter of d=16.46 cm and no corticomedullary differentiation, (C) being in arterial phase, (D) in portal phase, and (E, F) in delay phase].

On the 15 March 2023, the boy presented to the outpatient clinic with flank pain. Vital signs were within normal range and no significant unusual findings were noticed on the physical exam except for the skin lesions (Fig. 1). Creatinine level was tested and was normal at 0.71. Moreover, an abdomino-pelvic computed tomography (CT) scan with IV contrast was done and showed a single left kidney which had several nodular lesions with fat density which are typical for angiolipomatosis, besides, it showed enlarged kidney dimensions with no corticomedullary differentiation (Fig. 2). No signs of bleeding were reported and therefore the boy was only planned for surveillance for bleeding risk or malignant transformation.

## Discussion

The inherited neurocutaneous condition referred to as TSC is a disease characterised by pleomorphic features in numerous organ systems, including multiple benign hamartomas of the brain, eyes, heart, lung, liver, kidney, and skin^2,8^. TSC is an autosomal dominant disorder with a high penetrance, but new mutations are identified in ~70% of TSC cases^1^. The disease is believed to originate from inactivating mutations of TSC1 and TSC2, which are tumour suppressor genes located on chromosomes 9q34 and 16p13.3, respectively^1^.

Regarding the manifestations of TSC, there are significant differences in both the frequency and severity among individuals, whether related or not^9,10^. Most TSC patients have one or more of the typical skin lesions associated with the condition^2^. These lesions range from angiofibromas, fibrous plaques, collagenomas, periungual fibromas, gingival fibromas, hypopigmented macules, café au lait spots, to small molluscums^2^. On the other hand, seizures are the most frequent neurological finding, with epilepsy being a significant contributor to the disease morbidity^1^. Approximately 90% of children with TSC exhibit subependymal nodules and cortical glioneuronal hamartomas on brain magnetic resonance imaging^3,4,11^. These cortical glioneuronal hamartomas and pathogenic TSC2 mutations are risk factors for the onset of epilepsy^12^, which was evident in our case as the boy started having refractory myoclonic seizures which lead to diagnosis following an MRI imaging. Intellectual retardation has also been associated with a higher frequency and worse severity of epilepsy onset before the age of two^13^. Additionally, up to 65% of TSC patients, including the patient mentioned in our case, experience some degree of cognitive dysfunction^4,14^ Furthermore, more than 50% of TSC patients have cardiac rhabdomyomas, which can cause flow irregularities when they enlarge and restrict blood flow^15,16^. It is also crucial to monitor renal manifestations as they are the leading cause of death in TSC patients, particularly in younger individuals due to malignant transformation of renal angiomyolipomas into renal cell carcinoma^17^. Renal cystic disease is the second most frequent renal manifestation of TSC after angiomyolipomas^18,19^, and both have been detected in our case.

The diagnosis of Bourneville disease is primarily based on clinical findings with the aid of radiological imaging modalities, following criteria of major and minor findings^5^. Renal angiomyolipomas constitute a major feature, while multiple renal cysts constitute a minor one^5^. An early diagnosis leads to early management planning, which, in turn, leads to better outcomes. Treatment should be coordinated among different experts involved in relevant specialties, with regular imaging evaluations. In our case, as soon as the angiolipomatosis lesions were reported, their size had to be identified. For sporadic lesions, a size of less than 6 cm confirms a low risk of malignant transformation, and a surveillance-only management plan is followed. Surveillance consists of performing Uroscan after 6 and 12 months, and then yearly for 5 years, using the same imaging modality. If no additional lesions or increase in size are noted, surveillance via kidney ultrasound every 3 years is deemed sufficient^20^. Other assessment tools include electroencephalogram, retinal evaluation, skin examination, electrocardiography, echocardiography, and neurodevelopmental assessment tests involving brain, heart, and skin evaluation^7^. In our case, symptomatic treatment was also required to control seizures, and therefore, antiepileptic drugs were initiated. Other treatment modalities are often considered for cosmetic significance and include excision, dermabrasion, and laser treatment to control cutaneous symptoms^7^. However, the remarkable aspect in the treatment of our case is that despite the presence of acute renal findings on the Uroscan and a congenital single kidney, there has been no need for hospitalisation or significant renal symptoms throughout the 14-year course of the disease. This highlights the importance of regular surveillance and management, as well as the potential strength of the remaining kidney function in adapting to the changes posed by TSC.

## Conclusion

In conclusion, this case, which has been reported in line with SCARE criteria^21^, presents a patient with TSC. Diagnosis of such disease is mainly clinical and treatment requires a multidisciplinary approach. Early diagnosis, proper surveillance, and coordinated management are crucial for optimal outcomes. This case report highlights TSC in a patient who was never hospitalised for his condition despite having a congenital single kidney, a rare finding which makes the disease more complicated. Further research and awareness are needed for better understanding and management of TSC in diverse populations.

## Ethical approval

Ethical approval was obtained from the IRB of Bahman hospital.

## Consent

Patient’s guardian voluntary consent for the publication of this case and images was taken while keeping patient identity anonymous. Consent was also obtained from all other involved parties including attending physicians and radiologist following the discussed case.

## Source of funding

Not applicable.

## Author contribution

W.B. and M.K.: contributed equally to the work in data curation, writing—original draft, writing—review and editing. O.R.: supervision, writing—review, validation of final review.

## Conflicts of interest disclosure

The authors of this article declare no conflicts of interest.

## Research registration unique identifying number (UIN)

Not applicable.

## Guarantor

Wafaa Bzeih, Mohamad Kbar, Oussama Rihan.

## Data availability statement

Data sharing is available upon request.

## Provenance and peer review

The paper was not invited nor published.
